# Two-Dimensional Analysis of Digital Images through Vector Graphic Editors in Dentistry: New Calibration and Analysis Protocol Based on a Scoping Review

**DOI:** 10.3390/ijerph18094497

**Published:** 2021-04-23

**Authors:** Samuel Rodríguez-López, Matías Ferrán Escobedo Martínez, Luis Junquera, María García-Pola

**Affiliations:** 1 Department of Operative Dentistry, School of Dentistry, University of Oviedo, C/. Catedrático Serrano s/n., 33006 Oviedo, Spain; escobedomatias@uniovi.es; 2 Department of Oral and Maxillofacial Surgery and Oral Medicine, School of Dentistry, University of Oviedo, C/. Catedrático Serrano s/n., 33006 Oviedo, Spain; junquera@uniovi.es (L.J.); mjgarcia@uniovi.es (M.G.-P.)

**Keywords:** vector graphics editor, two-dimensional analysis, photographic analysis protocol, Adobe Illustrator, CorelDRAW, Inkscape

## Abstract

This review was carried out to analyse the functions of three Vector Graphic Editor applications (VGEs) applicable to clinical or research practice, and through this we propose a two-dimensional image analysis protocol in a VGE. We adapted the review method from the PRISMA-ScR protocol. Pubmed, Embase, Web of Science, and Scopus were searched until June 2020 with the following keywords: Vector Graphics Editor, Vector Graphics Editor Dentistry, Adobe Illustrator, Adobe Illustrator Dentistry, Coreldraw, Coreldraw Dentistry, Inkscape, Inkscape Dentistry. The publications found described the functions of the following VGEs: Adobe Illustrator, CorelDRAW, and Inkscape. The possibility of replicating the procedures to perform the VGE functions was analysed using each study’s data. The search yielded 1032 publications. After the selection, 21 articles met the eligibility criteria. They described eight VGE functions: line tracing, landmarks tracing, linear measurement recording, angular measurement recording, image calibration, image overlay, file transfer, and vector graphics development. The features offered by the VGEs bring great precision and objectivity to two-dimensional image analysis. The image analysis and editing procedures are currently not protocolised. Thus, a protocol for image calibration and measurement recording is proposed in order to guarantee the protocol’s replication.

## 1. Introduction

Vector Graphic Editors (VGEs) are software programmes developed in graphic design to edit and create images. The appearance of these tools determined the beginning of “computer-aided graphic design”, which involved implementing CAD-CAM (Computer-Aided Design–Computer-Aided Manufacturing) in illustration, signage, editorial printing, fashion design, and multimedia. VGEs enable one to assess, measure, and modify the digital project’s dimensions to adapt it to the physical format it will have [1,2]. Three of the most used VGEs are Adobe Illustrator, CorelDRAW, and Inkscape. The first to be commercialised was Adobe Illustrator version 1.0 in 1987. Illustrator was only for Macintosh until 1989 [3,4,5], when a version for PC was launched, coinciding with the appearance of CorelDRAW version 1.0 [4,6,7], an application aimed mainly at PCs [2]. Inkscape was the last to be launched in 2003, and unlike the other two, is free open source software [4,8].

These programmes are based on mathematical systems such as the Bezier curve and produce images called vector graphics (VG) or object-oriented images, formed by a series of joined points called nodes [3,9,10]. This differentiates VGEs from raster graphics editors such as Adobe Photoshop and Corel Paint, which work with bitmap images formed by pixels [1,2,3,4,11]. The graphic elements present in a vector file are named “objects”, and each one has a complete entity with properties such as colour, form, size, and position on the screen, all included in its mathematical definition [1,2,3]. In vector graphics, an object can be manipulated by changing its properties successively without losing its quality or affecting the other objects in the image. VGEs have certain advantages that justify their use in graphic design and their usefulness in other disciplines, such as biomedical sciences. These advantages are inherent in the characteristics of vector graphics, namely, their scalability; (i.e., their size can be changed without loss of image quality); their small size compared to bitmap files; and their ability to have different formats such as EPS, PDF, WFL, and SVG, which make vector graphics compatible and versatile to work with other software [1,2,7,12]. Once the vector graphic is generated and saved in different formats, the most used being Scalable Vector Graphics (SVG) or Portable Document Format (PDF), the file can be reopened, even with a different VGE, and the objects that compose it will keep being independent and modifiable. Additionally, the objects in the file remain independent, enabling one to modify them or even to add new objects without loss of quality [13,14,15].

In dentistry, the two-dimensional analysis of photographs, radiological tests, or other images is paramount for diagnosis and treatment planning in clinical practice and research. Implementing the functions offered by the VGEs can be advantageous to reduce work time or provide more objective analyses. Among these functions is the compatibility between programmes, the linear and angular measurement records, image superposition, the development of digital illustrations [10,16], and the calibration of images to create scale, that is, to establish a mathematical relationship between the dimensions of an object and those that appear in its image on the screen [2,17,18].

The purpose of this review was to analyse the functions of three VGEs described in the literature applied to clinical and research practice. Through this analysis, a two-dimensional analysis protocol of images was created and described in order to serve as a basis for further research and practice.

## 2. Materials and Methods

### 2.1. Protocol

This section was planned following the PRISMA-ScR Protocol (Preferred Reporting Items for Systematic Reviews and Meta-analyses: Extension for Scoping Reviews) [19].

### 2.2. Focused Question

Do Vector Graphic Editors have application in clinical practice and/or dental research?

### 2.3. Eligibility Criteria

The following eligibility criteria were considered: Studies that describe in their methodological section a function of the following Vector Graphic Editors: Adobe Illustrator, CorelDRAW, or Inkscape.

#### 2.3.1. Type of Study

There were no restraints regarding the type of study.

#### 2.3.2. Language

The selected articles had to be written in English or Spanish as primary sources, and a second language was also allowed.

#### 2.3.3. Years Considered

There were no restraints regarding the publication dates of the works.

Comments or letters, patents, posters, lectures, and narrative reviews were considered as exclusion criteria.

### 2.4. Information Sources

The search was developed under the electronic system of the following sources: PubMed, Embase, Web of Science, and Scopus. The search was initiated on 22 January and ended on 27 May 2020. Search. The following keywords were included in the strategy: Vector Graphics Editor, Vector Graphics Editor Dentistry, Adobe Illustrator, Adobe Illustrator Dentistry, Coreldraw, Coreldraw Dentistry, Inkscape, Inkscape Dentistry.

### 2.5. Study Selection and Data Collection Process

Duplicated articles from different sources were detected, and duplicates removed. Subsequently and independently, two authors (R-L and G-P) carried out the selection of the articles that met the inclusion criteria based on the title, abstract, or full text if necessary. A third reviewer (F-V) intervened when there was no agreement between the first two. The articles that fulfilled the eligibility criteria were selected for full-text review.

### 2.6. Data Items

For each study, the name of the first author, the year of publication, and the objectives were listed. Additionally, the following variables were considered: country in which the study was carried out, dental specialty of the publication, VGE used, image type on which the analysis was performed, study design and VGE functions, considering the image analysis and editing procedures performed with a VGE. The procedures were grouped into eight terms according to their functions: “Trace Landmarks”, “Trace Lines”, “Linear Measurements”, “Angular Measurements”, “Overlap Images”, and “Image Calibration”, to establish a scale or position relationship, using an object with known dimensions or other references. “Vector graphics development” was to carry out work with a vector graphic, and “File transfer” was for the transfer and compatibility of files between different programmes. In addition, to determine if it was possible to replicate the measurement recording and image calibration protocols used, all the data provided by each study in the text, images, and figures, as well as in the Supplementary Materials, if any, were analysed. To classify the level of precision in the description of the procedures carried out on the VGEs, four degrees were set: the first one includes the studies that cite the functions performed without enabling the replication of the procedures; the second contains the studies that provide explanations or images of the procedures without guaranteeing their replication; the third consists of those studies that provide explanations and images of the procedures carried out, making it possible to replicate a large part of the protocol steps; and the fourth consists of those with a level of precision in the explanation of the procedures that ensures their reproducibility.

### 2.7. Synthesis of Results

The results were reached through the descriptive analysis of the variables.

## 3. Results

### 3.1. Study Selection

**Figure 1:**
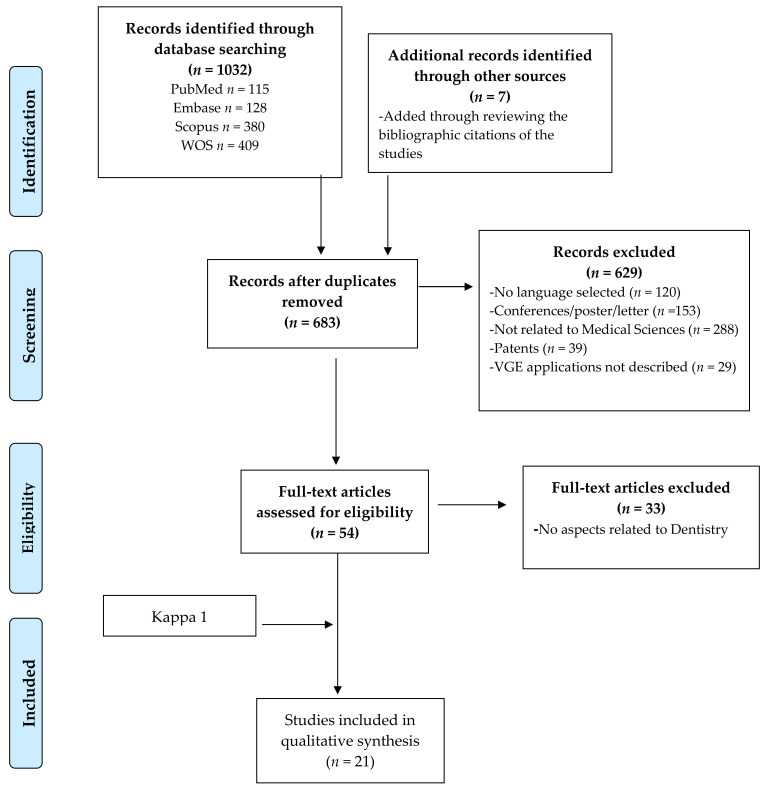
Flow diagram with the information through the phases of study selection based on PRISMA (Moher et al., 2009) [19].

PubMed provided 115 articles (1989–February 2020), Embase yielded 128 (1991–March 2020), Scopus brought 380 (1989–April 2020), and Web of Science 409 (1986–February 2019), forming a total of 1032 articles. After removing duplicates (*n* = 356) using the title and author(s), the sample was reduced to 676 articles. The remaining selection steps are depicted in Figure 1.

From all the samples, 47 dealt with biomedical sciences and described VGEs functions. A total of 33 that did not deal with aspects related to dentistry were excluded. After reading the full text and reviewing the bibliography, 21 publications were selected for the preparation of this review, with a 100% agreement of the reviewers (R-L and G-P) (Kappa Index = 1).

The selected papers date between 2006 and 2019 [20,21,22,23,24,25,26,27,28,29,30,31,32,33,34,35,36,37,38,39,40], are written in English, and are from the following countries: Brazil (*n* = 8), Bulgary (*n* = 1), Canada (*n* = 1), China (*n* = 1), Estonia (*n* = 1), Japan (*n* = 1), Jordan (*n* = 1), India (*n* = 2), Poland (*n* = 1), Saudi Arabia (*n* = 1), and Turkey (*n* = 3). Supplemental Table S1 shows the results of the variables analysed in the selected papers.

### 3.2. Study Characteristics

The registered studies were: in vitro study (*n* = 4) [22,23,29,31], fine element study (*n* = 1) [27], cross-sectional study (*n* = 9) [20,21,25,30,32,34,35,37,39], case–control (*n* = 4) [24,28,33,36], and cohort study (*n* = 3) [26,38,40].

The dental specialties covered in the research were: endodontics (*n* = 3) [22,23,31], restorative dentistry (*n* = 3) [27,33,36], orthodontics (*n* = 8) [24,25,29,30,32,37,38,39], and prosthodontics (*n* = 7) [20,21,26,28,34,35,40].

#### 3.2.1. Types of VGEs Analysed

CorelDRAW (Corel Corp and Coral Ltd., Ottawa, ON, Canada) (*n* = 19), in its different versions, was the most used VGE among the included works [20,21,22,23,24,26,27,28,29,30,31,32,33,34,35,36,38,39,40]. Adobe Illustrator (Adobe Systems Inc, San Jose, CA, USA) was used in two studies [25,37], and none of the selected articles described applications with Inkscape.

#### 3.2.2. Types of Images Analysed

Radiological images were the most frequently used, such as: periapical radiographs (*n* = 7) [22,23,26,33,36,38,40], cross-sectional images of CBCT (Cone Beam Computed Tomography) (*n* = 3) [27,31,39], cephalometric radiographs (*n* = 2) [24,25], and panoramic radiographs (*n* = 3) [26,28,40]. The second-most used source of images was standardised photographs of the smile (*n* = 3) [20,21,35], face (*n* = 1) [34], and dental casts or typodonts (*n* = 2) [29,37]. Paranhons et al. in two included publications used 3D dental model images from STL files (Standard Triangle Language) (*n* = 2) [30,32].

#### 3.2.3. VGEs Functions

The trace landmark functions (*n* = 6) [22,24,30,32,34,39] and reference lines (*n* = 13) [20,21,22,23,24,25,30,32,33,36,37,38,39] on key anatomical structures, along with image calibration (*n* = 8) [26,28,31,34,37,38,39,40], facilitate and improve the accuracy of other functions such as image overlay or measurement recording.

Overlapping images (*n* = 4) were used to compare the studied areas before and after treatments or experimental interventions. In the works of Constante et al. and Gianastasio et al., in vitro effects of different root canal instrumentation techniques were evaluated [22,23,31]. Menon et al. compared images before and after pulp coatings [36], and Liu et al. compared cephalometrics before and after orthodontic treatment [24].

In the two-dimensional analysis of images, linear measurement (*n* = 16) [20,21,22,23,24,25,26,28,33,34,35,36,37,38,39,40] or angular measurement (*n* = 4) [22,24,29,39] can be recorded. The review of the selected articles, grouped according to their specialty, provided the following information on the application of these linear and angular measurements. In endodontics, it was the analysis of the alterations that occur in the root canals after practicing different instrumentation techniques and analysis of the influence of the curvature of the canal in the effectiveness of different instrumentation techniques [22,23]. In restorative dentistry, it was the quantification of secondary dentin formation on periapical radiographs after performing pulp coverings [33,36]. In prosthodontics, it was the recording on photographs of the necessary measurements to calculate the aesthetic dental [20,21,35] and facial [34] proportions and to quantify crestal bone loss around implants through radiographs [26,28,40]. In orthodontics, it was the calculation of the orientation and position of the lower incisors in photographs and thus analysis of the influence of certain types of arches on the alignment of teeth [29], and in CBCT cuts it was the analysis of their influence on the level of the alveolar bone [39]. Liu et al. recorded the measurements resulting from cephalometric tracings to contrast the effects of a treatment technique [24]. Nassif et al. quantified root resorption after applying two orthodontic techniques [38]. Disthaporn et al. recorded measurements from dental cast photographs to analyse dental positions in patients and occlusal relationships in patients with repaired unilateral cleft of the lip and palate [37], and Nomura et al. measured the position of the lower lip in facial profiles outlined from cephalographies [25].

The development of vector graphics (*n* = 4), was performed to generate a cephalometric tracing template [24] to delineate the contour of a tooth on a CBCT section [27] that served as the basis for a finite element model or to generate illustrations for surveys [25,30].

File compatibility (*n* = 7) to complement the functions of different software was used by Constante et al. for recording measurements in radiographs using AutoCAD and CorelDRAW [23]. Disthaporn et al. modified the photo formats through Photoshop to work in Illustrator [37]. Gianastasi et al. [31] calibrated CBCT cuts from iCAT in CorelDRAW to transfer them to Photoshop. Manchorova-Velev et al. generated a vector graphic with CorelDRAW and transferred it to a finite element model engineering programme [27]. Paranhons et al. translated images from 3D scan models into CorelDRAW for editing [30,32], and Srebrzyńska-Witek et al. transferred CBCT cuts from the iCAT view to Irfan Viewer to calibrate the images and record measurements in CorelDRAW [39].

#### 3.2.4. The Classification of the Precision Level in the Description of the Procedures to Record Measurements and Image Calibration in the VGEs

Among the selected works, five cite the functions performed with the VGEs in their methodological section, without providing explanations that make possible the replication of the protocol followed [22,24,25,26,40]. Nine provide explanations or images to facilitate the understanding of the procedures that were carried out to conduct the functions in the VGEs [20,21,28,31,33,34,35,36,38], and four provide explanations and images detailing many of the procedures carried out [23,37,39]. Nevertheless, the reader should interpret some steps in order to follow the protocol carried out in these studies. The description of the protocol is undertaken in the next section.

## 4. Protocol Description of a Two-Dimensional Image Analysis

The following protocol details the steps to follow in a VGE to perform a two-dimensional analysis of an image calibrated from an object with standardised dimensions included in a photograph, thus establishing a scale. The example shows the photograph of a smile, taken according to the American Academy of Cosmetic Dentistry guide’s protocol [41]. To calibrate the image, a rectangular adhesive calibrated at 1 cm, used as a tab for scale, was placed with an incorrect orientation to explain how to correct tab displacement in the VGE. The objective was to measure the dimensions of the teeth from a frontal view.

**Figure 2:**
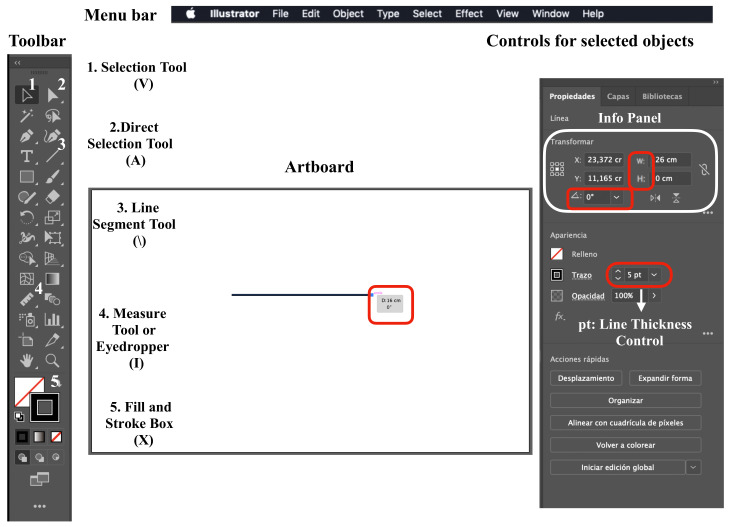
Adobe Illustrator workspace. (V): selection tool command, (A): direct selection tool command, (\) line segment tool command, (I): measure tool or eyedropper command, (X): fill and stroke box command to change color, (W): linear segmentlLength, pt: points that compose the linear segment thickness.

**Figure 3:**
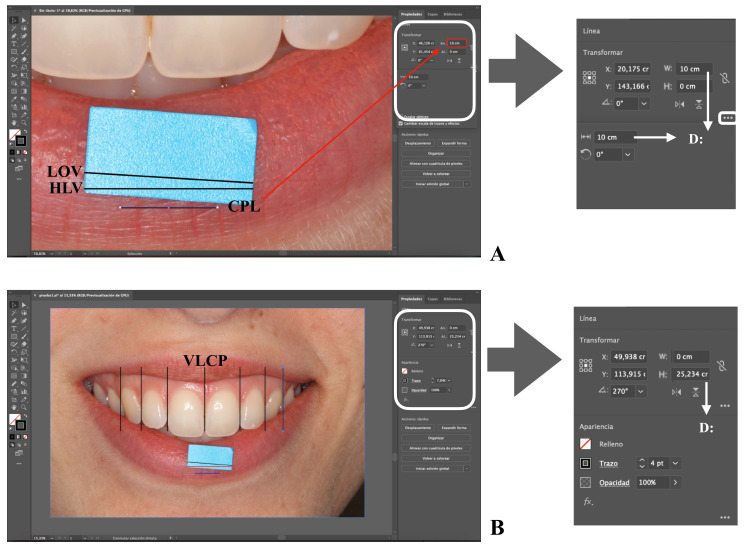
Screenshots of the sample image with the different steps of the protocol. LOV: line on the verifying witness. HLV: horizontal line on the verifying tab for scale. CPL: calibrated parallel horizontal line. VLVP: vertical line over contact point. D: distance. Screenshots of the sample image with the different steps of the protocol (**A**,**B**).

**Figure 4:**
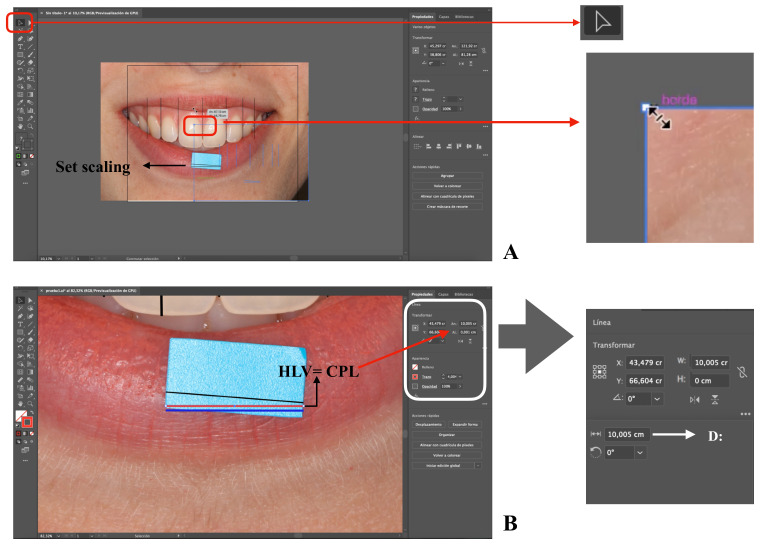
Screenshots of the sample image with the different steps of the protocol. LOV: line on the verifying witness. HLV: horizontal line on the verifying tab for scale. CPL: calibrated parallel horizontal line. D: distance. Screenshots of the sample image with the different steps of the protocol (**A**,**B**).

**Figure 5:**
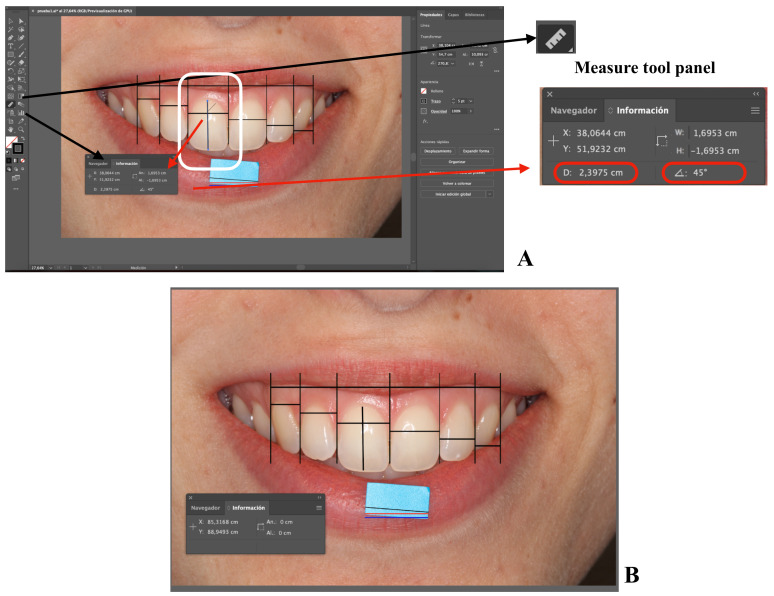
Screenshots of the sample image with the different steps of the protocol. Screenshots of the sample image with the different steps of the protocol (**A**,**B**).

In Figure 2, the components of the workspace (programme interface) are graphically represented: the artboard (digital representation of an area in reality), menu bar, tools bar, and the controls for the selected object bar. Only those functions that are used in this protocol are indicated. In Figure 3, Figure 4 and Figure 5 are the screenshots of the example with the different steps described below, and in Table 1 the computer typing necessary to follow the protocol (Steps) is detailed (Video 1 shows all the procedures described below).

The programme works with a coordinate system (X and Y), where the positions occupied by the objects on the artboard are defined. The information of the position of the objects appears in the Info Panel (Figure 2), where the parameters Width (W) and Height (H) are related to X and Y, respectively. There is also the parameter angulation (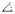) with respect to the artboard. To make linear measurements, the parameter Distance (D) is used, and it has the same value as W, when H has a value of 0 and the angular component is 0° or 180°. Additionally, it has the same value as H when W is 0 and the angular component is 90° or 270°. To consult D, an object is selected and the selection tool is positioned in one of the ends of the object (V), on expanding the Info Panel by clicking on the three points in the lower-left corner (Figure 2 and Figure 3), or on the measure tool panel (Figure 5) [42].

The first step is to create a new artboard. Open Adobe Illustrator CC (Adobe Systems Inc, San Jose, CA, USA) and on the Start screen click on Create new. Once opened, on the right side of the screen in the preset details menu, select the artboard dimensions, orientation, and measurement units. To set the artboard dimensions, consider whether the file is planned to be transferred to other software and/or the type of rendering (such as printing) of the project. In the example provided, units in centimetres (cm) and a landscape orientation were chosen.

Once the new blank artboard is created, the image to be worked on via the Embedded artwork mode is uploaded (Table 1, Step 1) (Figure 2) [43]. The first line is traced with the appropriate orientation, occupying the entire extension of the tab for scale. This line is called the Line on the verifying tab (LOV) (Table 1, Step 2) (Figure 3A). If corrections are needed, the steps to be followed are described in Table 1: Step 3, 4, and 5. The second step is to draw a parallel of LOV, called the horizontal line on the verifying tab (HLV) (Table 1, Step 6) (Figure 3A), and its orientation will be corrected by setting the parameters 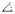 and H to 0. 

The third step is to draw a parallel to HLV, which is referred to as the calibrated parallel horizontal line (CPL) (Table 1, Step 6) (Figure 3A). With CPL selected, a length (D) of 1 cm is settled by modifying the W parameter. To facilitate the calibration work, it is recommended to work with a CLP of 10 cm. This way, the image size will not be excessively reduced and the zoom control and mouse movements will be improved (Figure 3A). Nevertheless, by resizing the CPL, the obtained scale will change from 1:1 to 10:1. In the case of working with a CPL of 10 cm, it is recommended to adopt a line thickness of 3 or 4 points (pt) (Figure 2); on the other hand, for a 10-mm CPL, the recommended thickness is 0.75–1-pt lines. The choice of thickness facilitates their drawing on the image and does not influence the measurements since Illustrator measures from the center of the line. Therefore, each line is an *object* and is mathematically defined in the software.

The next step is to mark the points where the central zone of the proximal contact area between the two teeth is located. This is performed by drawing vertical lines, which are called the vertical line over contact poin” (VLCP) (Table 1, Step 7) (Figure 3B). Next, the HLV image and all of the VLCP lines, except CLP, are selected (Table 1, Step 8). With the entire set selected, the mouse pointer is displaced from a corner of the image to proportionally resize it and the selected objects. (Table 1, Step 9) (Figure 4A). It will be observed that as the size decreases, the image is displaced towards a corner of the workspace. (Figure 4A). All of the selected sets will be resized to make both HLV and CPL lines with the same length. For this, W or D parameters will be consulted. It is possible that the set might be moved and the step repeated, since, once resizing, the references are displaced (Table 1, Step 9 and 10) (Figure 4B) [44]. It is recommended to vary the zoom, since by increasing it we will achieve more precise size modifications.

At the end of this procedure, the image will be calibrated according to the object with known dimensions in a 1:1 scale (in this example 10:1), and the measurements are related to the real dimensions of the element included in the image. It is important to mention that when the image is uploaded to Adobe Illustrator, it will appear in the workspace with its full resolution size [45,46]. The photograph is a bitmap image composed of pixels, and it loses quality when resized since the number of pixels is modified. Due to this, it is recommended to draw the lines before reducing the image size. When the measurements are between lines or landmarks with a VGE, the measurement is taken between independent objects; their position is mathematically determined, and they are not made up of pixels. The default Adobe Illustrator unit is points (pt), and a pt equals 0.3528 mm [47].

The measurement record can be carried out either by drawing new lines (Table 1, Step 7) (Figure 5A,B) or using the measure tool (Table 1, Step 11) (Figure 5A,B) [48]. This way, it is possible to take horizontal linear measurements such as the mesio-distal width of the teeth, measuring between two contiguous VLCPs, or the intercanine width, measuring the most distal VLCP from one side to its counterpart. Additionally, it is possible to take vertical linear measurements such as the gingivo-incisal length of a tooth from the gingival zenith to the incisal edge (Table 1, Step 7 and 11) (Figure 5A). The measure tool also allows one to record angular measurements (Table 1, Step 11) (Figure 5A).

On the other hand, there are several vector programmes developed to design in Engineering or Architecture, such as AutoCAD [49]. All of the editing and analysis functions described in this paper would be carried out in those programmes. This work describes the procedure in Adobe Illustrator due to its nature of image processing software, presenting a more straightforward interface for image tracing and scaling.

## 5. Discussion

The present scoping review of twenty-one scientific articles shows that the functions of the VGEs are useful for the development and collection of data in dental studies. These programmes were used in four dental specialities, the most frequent being orthodontics and prosthodontics (38% and 33%, respectively). The studies were carried out in 10 different countries, evidencing the high availability of these programmes. VGEs have been used for the development of VGs and file transfer in medicine since the 1990s [50,51,52,53]. However, measurement recording and image calibration were not developed until the 2000s [44,45], coinciding with the first dental studies [20,21].

In recent decades, three-dimensional analysis technologies such as CBCTs and 3D scanners have been incorporated into dentistry. This has represented a great advance in the diagnosis and planning of treatments [54,55,56]. In spite of this, the tests that allow a two-dimensional analysis are still valid in many cases [57,58] since they provide the most objective projection or, from a cost–benefit point of view, they are the most reasonable option due to the shorter registration times and radiation dose or because the equipment is simpler and more accessible [59,60,61].

The studies analysed in the area of dentistry that used VGEs have relied mainly on CorelDRAW. This can be justified in that this has been the most widespread for PC equipment [2]. In this review, no article describing procedures with Inkscape was found, although it is the programme with the easiest access since it is free and, therefore, can be used without the need to purchase any license [8]. Nevertheless, it is the most recent program, and it is not developed by any company that commercialises it.

The two-dimensional analyses of digital images in the research included in this review have been carried out essentially on radiological images (*n* = 13) [22,23,24,25,26,27,28,31,33,36,38,39,40] and photographs (*n* = 6) [20,21,34,35,37]. These images were calibrated and reference points and lines were drawn on them in order to record linear and angular measurements, that is, to collect the data necessary to perform the statistical analysis in the studies. 

The possibility of superimposing images allows for more extensive analyses since it is possible to carry out comparisons of images recorded before and after the intervention of the researcher. An example of this is the endodontic studies by Constante et al. [22,23] and Giannastasi et al. [31] or pulp response to aggressions by Menon et al. [36]. However, there are protocols describing procedures for overlapping images with clinical purposes in the medical literature, such as 3D CT images and fluoroscopies to facilitate catheterisation techniques in cardiology [62]. Another example is the work of Coobes et al. [54], who studied the overlapping of facial photographs to record the necessary measures for the evaluation and follow-up of patients who are candidates for oculoplasty.

The easy transfer of files and compatibility of the formats that VGEs support and generate allow the completion of the functions that these programmes have with other programmes. Among the selected articles, that by Srebrzyńska-Witek et al. [39] must be highlighted. In this work, the authors recorded the measurements in CBCT slices, which were then transferred from the 3D iCat viewer software to CorelDRAW. The authors justified this procedure because with the viewer software they could not record all the measurements they needed. Manchorova-Veleva and Neshka [27] also performed a file transfer of CBCT cuts, generating a VG outlining the tooth contour on the cuts to create a vector graphic compatible with finite element modeling software.

The main limits of this review derive from the heterogeneity of the studies and the lack of precise descriptions of the processes carried out to calibrate images and record measurements. Therefore, a reference protocol was not found, nor similar procedures to guarantee that the steps to apply the VGE functions are replicable. Consequently, it is possible that variations in the precision of the recorded measurements may arise between different studies using VGEs. Nevertheless, there are publications that describe in detail all the steps needed to create scientific illustrations with VGEs [10,11,16,63,64].

Due to the lack of a unified protocol to analyse images with VGEs and apply it to dental clinical practice and research, this work proposes a protocol with Adobe Illustrator to record measurements and calibrate images, resizing them from a reference object. The aim is to create a scale that facilitates image overlapping procedures, taking more precise measurements and facilitating the replication of procedures.

With the VGEs proposed in the protocol of the previous section, it was possible to calibrate the images by adjusting to two decimals. However, other factors that significantly influence the precision, such as the resolution and quality of the image or its standardisation, in addition to the capacity of the software must be considered. This is related to the recording device and the protocol followed during the image collection process. This means that parameters such as the distance and position of the sensor with the object, luminosity, or radiation must be taken into account. These, and other issues, are needed to ensure the least possible bias and that the vision or projections collected are equivalent in order to be comparable and analysable.

In summary, VGEs present a series of advantages for two-dimensional analysis compared to other types of programmes such as raster graphics editors (Photoshop, Paint, Corel Photo-Paint) that work with bitmaps and, therefore, measure pixels that may vary in number and size when resizing the image [3,65]. In the VGEs dealt with here, the work is carried out through a digital coordinate system in which the measurement is taken from the lines or reference points, measuring between *objects* mathematically defined both in position and size.

## 6. Conclusions

The present scoping review shows that VGEs are a useful and accessible tool for clinical professionals and researchers. The functions offered by these tools provide greater precision and objectivity to the two-dimensional analysis of images, namely, file transfer and compatibility between programmes, drawing lines or reference points, recording linear and angular measurements, and image calibration and overlaying.

### Clinical Relevance

The present view highlights and synthesises the different functions of the VGEs described in the literature that are useful for clinical practice and research. Given the limitations observed in this review, a protocol for image calibration and measurement recording is proposed. This protocol is applicable to photographic, radiological, and other images and can serve as a basis for other researchers who want to propose more complex two-dimensional analyses.

## Figures and Tables

**Table 1:** Adobe Illustrator functions described in the protocol. LOV: line on the verifying witness. HLV: horizontal line on the verifying witness. CPL: calibrated parallel horizontal line. VLVP: vertical line over contact point. Controls. (V): selection tool command. (A): direct selection tool command. (\): line segment tool command. (I): measure tool or eyedropper command.

Adobe Illustrator Functions Described in the Protocol			
Step:	Performance	Selected Tool	Procedure
1	Place (import) files in the artwork		Menu bar “File” > “Place” > select the file > deselect “Link” to embed in the artwork > “Click Place”.
2	Draw a line (LOV)	Line Segment Tool (\)	+Hold click + Drag mouse + Drop mouse.
3	Modify dimension of a line	Selection Tool(V)	+Hold click + Drag mouse + Drop mouse.
4	Move object	Direct Selection Tool (A) or Selection Tool (V)	+Hold click + Drag mouse + Drop mouse *Shift: to orientate in right angles (only Selection Tool).
5	Correct/Go back		Control + Z/Menu bar “Edit” + “Undo”.
6	Draw a parallel line (HLV/CPL/VLCP)	Selection Tool (V) or Direct Selection Tool (A)	+Hold click over the line (HLV) + Hold Alt + Hold Shift + Drag mouse up or down+ Drop mouse + release Alt and Shift.
7	Draw a perfect horizontal or vertical line (LOV/VLCP)	Line Segment Tool (\)	+Hold click + Hold Shift + Drag mouse horizontally/vertically + Drop mouse
8	Select multiple objects	Selection Tool (V)	+Hold Shift + Click over object or drag mouse.
9	Set scaling	Selection Tool (V)	Step 8 + Hold Shift + Drag mouse to a corner of the image + Reduction symbol appears + Drag mouse towards the center of the screen.
10	Set movement	Selection Tool (V)	Step 8+ Hold Shift + Drag mouse + Drop mouse.
11	Use the measure tool	Measure tool (I) *Double click on the Eyedropper Tool	+Hold click on point of origin + Drag mouse + Hold mouse over end point. *If we want to measure perfect horizontals or verticals or angles of 45°, 90°, 135°, 180° and successively use Shift

## Supplementary Materials

The following are available online at https://www.mdpi.com/article/10.3390/ijerph18094497/s1, Table S1: Applications of Vector Graphic Editors in dentistry described in the bibliography, Video S1: two-dimensional analysis protocol of a digital image. Using adobe illustrator.
