# Supplementary material for: Two-Dimensional Analysis of Digital Images through Vector Graphic Editors in Dentistry: New Calibration and Analysis Protocol Based on a Scoping Review
Source: Int J Environ Res Public Health. 2021 Apr 23;18(9):4497. doi: 10.3390/ijerph18094497 (PMC8122989; doi:10.3390/ijerph18094497)
Supplement: Supplementary file 1 [file ijerph-18-04497-s001.zip › ijerph-1188530 Supplementary.pdf]

Supplementary Table S1. Applications of Vector Graphic Editors in dentistry described in the bibliography. VGE: Vector Graphic Editor. CBCT: Cone Beam Computed Tomography. STL: Standard Triangle Language. *: Classification of the level of precision in the description of the procedures to record measurements and image calibration in the VGE's: +: functions are mentioned without enabling the replication of the procedures , ++: provide explanations or images of the the procedures without guaranteeing their replication, +++: provide explanations and images of the procedures carried out, making it possible to replicate a large part of the protocol steps, ++++: present a level of precision in the explanation of the procedures that ensures the reproducibility of the procedures. N.A: Not analyzed.

Applications of VGE in Dentistry:								
**Author, year**	**Country**	**Specialty**	**VGE**	**Summary of Objectives**	**Image Type**	**VGE Functions**	**Possibility of replicating the protocol ***	**Study design**
**Constante et al. 2007 [22]**	Brazil	Endodontics	CorelDRAW 10	To assess the radicular curve position and angulation influence among three instrumentation techniques.	**Periapical radiographs**	**-Trace Landmarks.** -**Trace lines** **-Linear measurements** -**Angular measurement**s **- Overlap images (pre-post)**	+++	In vitro study
**Constante et al. 2007 [23]**	Brazil	Endodontics	CorelDRAW10	To compare three instrumentation techniques considering their efficacy and non-desired anatomical alterations.	**Periapical radiographs**	**-File transfer (autocad to VGE)** **-Trace lines** **-Linear measurement**s **- Overlap images (pre-post)**	+++	In vitro study
**Giannastasio et al. 2013 [31]**	Brazil	Endodontics	CorelDRAW	To assess the apical transportation produced by two instrumentation techniques.	**Cross-sectional images of CBCT**	**-File transfer (iCAT vision to VGE to photoshop)** **-Image Calibration:** the position of the acrylic resin block with regard to the X and Y axes.	++	In vitro study
**Manchorova-Veleva. 2011 [27]**	Bulgary	Restorative Dentistry	CorelDRAW 7.0	To study the size of cavity wall deformation in eight class I and II defects after composite restoration.	**Cross-sectional images of CBCT**	**- Vector graphics development**: Contour delineation **-File transfer (VGE to SolidWorks)**	N.A	Finite element study
**George et al. 2015 [33]**	India	Restorative Dentistry	CorelDRAW 13	To evaluate the reparative dentin formation in indirect pulp treatment using two different	**Periapical radiographs**	**-Trace Lines**. **-Linear measurements**	++	Case-control

				materials in primary teeth over a period of 6 months.				
**Menon et al. 2016 [36]**	India	Restorative Dentistry	CorelDRAWX3	To evaluate the reparative dentin formation in indirect pulp treatment using two different materials in primary molars over a period of 6 months.	**Periapical radiographs**	**-Overlap images (pre-post):** With the pulp chamber floor and amelocementary union **-Trace lines** -**Linear measurements**	++	Case-control.
**Liu et al. 2009 [24]**	China	Orthodontics	CorelDRAW 11.0	To compare cephalometrically to treatment techniques in postpeak stage patients with class II division 1.	**Cephalometric radiographs**	-**Trace lines** **-Trace Landmarks** **-Linear measurements** **-Angular measurements** **-Overlap images (pre-post):** with a reference grid **- Vector graphics development;** Set up reference grid	+	Case-control
**Baratieri et al. 2012 [29]**	Brazil	Orthodontics	CorelDraw 13	To evaluate the influence of the shape and the length limitation of superelastic nickel-titanium archwires on lower incisors inclination during alignment and levelling.	**Standardized photographs**	-**Angular measurement**s	+	In vitro study
**Paranhos et al. 2012 [30]**	Brazil	Orthodontics	CorelDRAW x3	To evaluate the correlation between the morphology of the mandibular dental arch and the maxillary central incisor crown.	**Dental cast STL file images.**	**-File transfer 3D Viewer (3Shape) to VGE** **-Trace Landmarks** -**Trace lines** **- Vector graphics development:** contour delineation of a tooth.	N.A	Cross-sectional study
**Paranhos et al. 2014 [32]**	Brazil	Orthodontics	CorelDRAWx3	To investigate the association between the mandibular arch morphology and the facial type of Brazilian Caucasians with natural normal occlusion.	**Dental cast STL file images.**	**-File transfer 3D Viewer (3Shape) to VG** **-Trace Landmarks** -**Trace lines**	N.A	Cross-sectional study
**Nomura et al. 2015 [25]**	Japan	Orthodontics	Adobe Illustrator	To determine whether observer and patient sex and race or ethnicity determine esthetic preferences for lip positions.	**Cephalometric radiographs**	**-Vector graphics development:** Contour delineation of a facial profile	+	Cross-sectional study

**Disthaporn et al. 2017 [37]**	Canada	Orthodontics	Adobe Illustrator 4.0	To assess qualitatively and quantitatively, through the dental cast, the dental positions and occlusal relationships in patients with repaired complete unilateral cleft of the lip and palate.	**Standardized photographs**	-**Tracing lines**<br>**-Linear measurements**<br>**-File transfer (Photoshop to VGE)**<br>**-Image Calibration**: ruler with millimetre scale<br>-**Trace Lines**<br>-**Linear Measurements**.	+++	Cross-sectional study<br>.
**Nassif et al. 2017 [38]**	Brazil	Orthodontics	CorelDRAW X7	To compare the degree of apical root resorption of maxillary incisors through two orthodontic techniques.	**Periapical radiographs**	**-Image Calibration:** using the workspace dimensions.<br>**-Trace lines**<br>-**Linear measurements**	++	Cohort study
**Srebrzyńska-Witek et al. 2018 [39]**	Poland	Orthodontics	CorelDRAW 9	To evaluate the influence of the position of inferior incisors and canines on the dimensions of the cortical and spongious bone of the anterior mandibular alveolar process.	**Cross-sectional images of CBCT**	**-File transfer iCAT to IrfanView to VHE**<br>**-Image Calibration**.<br>**-Trace Landmarks**<br>-**Trace lines**<br>-**Linear measurements**<br>**-Angular measurements**	+++	Cross-sectional study
**De Castro et al. 2006 [20]**	Brazil	Prosthodontics	CorelDRAW 11	To assess the presence of dental proportions in the smile of a population sample.	**Standardized photographs**	**-Trace Lines**.<br>**-Linear measurements**	++	Cross-sectional study
**Fayyad et al. 2006 [21]**	Jordan	Prosthodontics	CorelDRAW 10	To assess the presence of dental proportions in the smile of a population sample.	**Standardized photographs**	**-Trace Lines**.<br>**-Linear measurements**	++	Cross-sectional study
**Bilhan et al. 2011 [26]**	Turkey	Prosthodontics	CorelDRAW 11.00	To assess the influence of attachment types on the marginal bone loss around dental implants supporting mandibular overdentures.	**Panoramic radiographs / Periapical radiographs**	**-Image Calibration**: with the implant platform.<br>-**Linear measurements**	+	Cohort study
**Mumcu et al. 2011 [28]**	Turkey	Prosthodontics	CorelDRAW11.0	The impact of the size of the implants, age and gender of patients, and the presence of cantilevers on marginal bone loss	**Panoramic radiographs. Periapical radiographs**	**-Image Calibration**: with the implant platform.<br>-**Linear measurements**	+	Cohort study

				surrounding implants with fixed restorations was evaluated over 36-month.				
**Nguyen et al., 2016 [34]**	Estonia	Prosthodontics	CorelDRAW x3	To evaluate proportions of frontal facial soft-tissues of Vietnamese females compared to the golden proportion (GP).	**Standardised photographs**	**-Image Calibration** with a ruler. **-Tracing Landmarks** -**Linear measurements**	++	Cross-sectional study
**Niranjan et al. 2016 [35]**	Saudi Arabia	Prosthodontics	CorelDRAW 11	To assess the presence of dental proportions in the smile of a population sample.	**Standardized photographs**	**-Linear measurements**	++	Cross-sectional study
**Mumcu and Beklen 2019 [40]**	Turkey	Prosthodontics	CorelDRAW 11.0	To examine whether smoking causes low or high MBL around implant-supported fixed or removable prostheses, after excluding other clinical factors.	**Panoramic radiographs**	**-Image Calibration**: with the implant platform. -**Linear measurements**	++	Case-control.
